# Die Marginalvene – nach wie vor eine seltene Entität: Fallserie von 16 Patienten

**DOI:** 10.1007/s00104-022-01648-1

**Published:** 2022-06-29

**Authors:** D. Liebetrau, R. Marnoto, Y. Goßlau, S. Zerwes, Franz Stangl, W. A. Wohlgemuth, A. Hyhlik-Dürr

**Affiliations:** 1 Gefäßchirurgie, Medizinische Fakultät, Universität Augsburg, Stenglinstr. 2, 86156 Augsburg, Deutschland; 2 Artemed Klinikum München Süd, Zentrum für Herz- und Gefäßchirurgie, Am Isarkanal 30, 81379 München, Deutschland; 3 Klinik für Diagnostische und Interventionelle Radiologie und Neuroradiologie, Stenglinstr. 2, 86156 Augsburg, Deutschland; 4 Universitätsklinik und Poliklinik für Radiologie, Universitätsklinikum Halle (Saale), Ernst-Grube-Str. 40, 06120 Halle (Saale), Deutschland; 5 Klinik für Gefäßchirurgie und endovaskuläre Chirurgie, Universitätsklinikum Augsburg, Stenglinstr. 2, 86156 Augsburg, Deutschland

**Keywords:** AV-Malformation, Therapie, Outcome, Chirurgische Behandlung, Endovenöse Behandlung, Arteriovenous malformation, Therapy, Outcome, Surgical treatment, Endovenous treatment

## Abstract

**Hintergrund:**

Die Marginalvene (MV) ist eine angeborene, vorwiegend venöse Gefäßmalformation, die auf einer fehlenden Rückbildung des embryonalen Venensystems an den unteren Extremitäten beruht. Sie geht mit einer Vielzahl an Komplikationen einher. Bisher werden in der Literatur keine einheitlichen Therapieregime beschrieben.

**Fragestellung:**

Welche Behandlungsstrategien und Ergebnisse gibt es bei Patienten mit MV?

**Material und Methoden:**

Im Zeitraum 01.01.2008 bis 31.12.2020 wurden alle am Universitätsklinikum Augsburg behandelten Patienten mit Marginalvene retrospektiv aufgearbeitet.

**Ergebnisse:**

Das mediane Alter zum Diagnosezeitpunkt lag bei 14,8 Jahren (3–42 Jahre). 12/16 Patienten hatten eine Beinlängendifferenz. 75 % der Patienten (12/16) hatten bereits zur Diagnosestellung MV eine chronisch-venöse Insuffizienz (CVI). Im untersuchten Kollektiv wurden 62,5 % (10/16) der Patienten zum Zeitpunkt der Diagnosestellung mittels Kompression konservativ behandelt. Bei weiteren 31,3 % (5/16) der Patienten erfolgte primär eine offen-chirurgische Entfernung der MV und bei 1/16 Patienten wurde die MV primär mittels endovenöser Lasertherapie (EVLT) verschlossen; 15/16 Patienten wurden sekundär therapiert. 2,6 ± 2,4 (MW ± SD) Sekundärprozeduren wurden pro Patient im Follow up durchgeführt. Das mittlere Follow-up lag bei 8,1 Jahren.

**Diskussion:**

Zur Prävention/Vermeidung einer Progression einer CVI und Thrombembolieprophylaxe sollte nach Diagnosestellung die MV zeitnah verschlossen/entfernt werden. Die Anwendung chirurgisch-konventioneller Techniken zur Entfernung der MV scheint gegenüber der Behandlung mit minimal-invasiven Prozeduren hinsichtlich der Anzahl der erforderlichen Sekundäreingriffe von Vorteil.

## Abstract (en)

**Background:**

The marginal vein (MV) is a congenital, predominantly venous vascular malformation, which is based on a lack of regression of the embryonic venous system in the lower extremities and is associated with a variety of complications. So far, no uniform treatment regimens have been described in the literature.

**Objective:**

What are the treatment strategies and outcomes in patients with a MV?

**Material and methods:**

In the period from 1 January 2008 to 31 December 2020, all patients treated at the University Hospital Augsburg with MV were retrospectively reviewed.

**Results:**

The median age at the time of diagnosis was 14.8 years (3–42 years). Out of 16 patients 12 had a leg length difference, 75% of patients (12/16) already had chronic venous insufficiency (CVI) at the time of diagnosis of MV. Open surgical removal of MV was performed as first-line treatment in 31.3% (5/16) patients. The MV was primarily closed by endovenous laser therapy (EVLT) in 1/16 patients, 15/16 patients were treated with redo procedures and 2.6 ± 2.4 (mean±SD) redo procedures were performed per patient in follow-up. The mean follow-up was 8.1 years.

**Discussion:**

In order to prevent/avoid progression of CVI and thrombosis prophylaxis, the MV should be closed/removed promptly after diagnosis. The use of conventional surgical techniques for the removal of MV seems to have an advantage over treatment with minimally invasive procedures in terms of the number of secondary interventions required.

## Hintergrund

Die Marginalvene (MV) ist eine angeborene, vorwiegend venöse Gefäßmalformation, die auf einer fehlenden Rückbildung des embryonalen Venensystems an den unteren Extremitäten beruht [9]. Sie verläuft an der lateralen Seite der unteren Extremität, besitzt in der Regel keine Venenklappen und mündet in verschiedenen Etagen in das tiefe Venensystem [22]. Entsprechend des venösen In- und Outflows kann die Vene klassifiziert werden. Dabei kann das tiefe Venensystem regelrecht, hypoplastisch oder sogar aplastisch ausgebildet sein [26]. Das Vorhandensein einer Marginalvene ist häufig kombiniert mit anderen Syndromen. So wird die Marginalvene vermehrt bei Patienten mit einem Klippel-Trénaunay-Syndrom (KTS) beobachtet [16, 27].

Weiterhin treten vermehrt arteriovenöse (AV‑)Fisteln in Kombination mit einer MV auf [9], sodass es bei zusätzlicher Aplasie von Venenklappen zu einer rasch fortschreitenden chronisch-venösen Insuffizienz (CVI) mit den entsprechenden Folgeschäden kommen kann [9].

## Zielsetzung der Arbeit

Es besteht aktuell kein Konsens über die Vorgehensweise zur Therapie der MV [8, 19]. Daher ist das Ziel dieser Untersuchung die Evaluation verschiedener Behandlungsstrategien und deren Ergebnis im Langzeitverlauf sowie die Erstellung eines Therapiealgorithmus zur Behandlung der MV.

## Patientenkollektiv und Methoden

Im Zeitraum 01.01.2008 bis 31.12.2020 wurden alle am Universitätsklinikum Augsburg behandelten Patienten mit Marginalvene in einer Datenbank (Microsoft-Excel®-Version 2019, Redmond, WA, USA) erfasst und retrospektiv ausgewertet. Eingeschlossen wurden alle Patienten, die sich mit einer dokumentierten Marginalvene vorstellten. Es konnten 16 Fälle (8 männliche, 8 weibliche Patienten) nach Anwendung der Einschlusskriterien in die Datenerhebung eingeschlossen werden.

Ausgewertet wurden Diagnosen und Nebendiagnosen, Symptomatik, Charakteristik der Marginalvene, Zeitpunkt der Diagnosestellung, Therapiemethode sowie deren Komplikationen und Therapieergebnisse im Verlauf. Als Therapieergebnis wurden der komplette Verschluss und oder die komplette Entfernung der Marginalvene, die Verbesserung der Beschwerden (Schmerzen, Juckreiz, Schwellung, Abheilung des Ulkus), das kosmetische Ergebnis sowie die Zufriedenheit der Patienten erhoben.

Die Diagnosestellung erfolgte mittels farbkodierter Duplexsonographie (FKDS), Magnetresonanz(MR)-Phlebographie oder konventioneller Phlebographie. Alle Patienten wurden zur Nachkontrolle einbestellt oder über einen standardisierten Fragebogen telefonisch über ihren Zustand befragt.

## Ergebnisse

**Figure 1:**
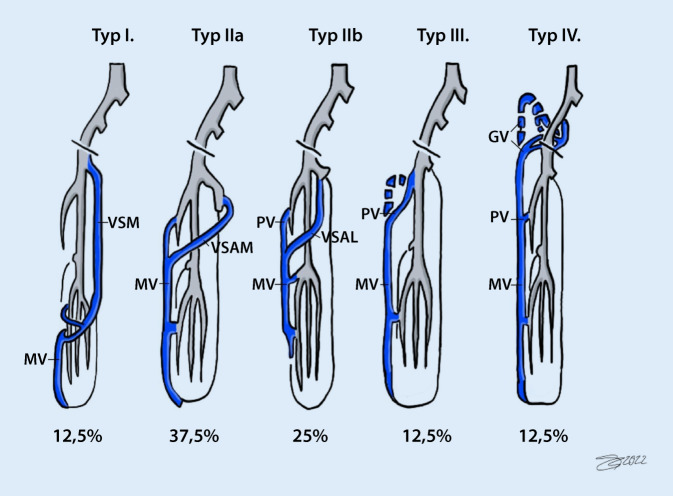

Das mediane Alter zum Diagnosezeitpunkt lag bei 14,8 Jahren (3–42 Jahre). Bei 50 % der Patienten trat die MV kombiniert mit anderen Syndromen auf. Zwölf der 16 Patienten hatten eine Beinlängendifferenz (Tab. 1). 75 % der Patienten (12/16) hatten bereits zur Diagnosestellung MV eine chronisch-venöse Insuffizienz (CVI) und 2 der 16 Patienten eine arteriovenöse (AV‑)Fistel (Tab. 2). Gemäß der Klassifikation nach Weber [26] war der Typ II (62 %) der häufigste Typ (Abb. 1).

**Table 1:** 

Patientencharakteristika	Gesamt	
	(*n*)	(%)
**Patientenkollektiv**	**16**	**100,0**
Geschlecht (M/W)	**8/8**	50/50
Alter (MW ± SD) bei Diagnose	16,8 ± 10,62	
Medianes Alter in Jahren bei Diagnose	14,8	
**Stadium der Marginalvene**		
*Weber-Stadium*	**16**	100,0
I	**2**	12,5
IIa	**6**	37,5
IIb	**4**	25,0
III	**2**	12,5
IV	**2**	12,5
**Anlage der Marginalvene**		
*Isoliert*	**8**	50,0
*Kombiniert mit anderen Syndromen*	**8**	50,0
KTS	**2**	12,5
ClOVES	**2**	12,5
BRBNS-Syndrom	**2**	12,5
Proteus-Syndrom	**1**	6,3
CMTC Syndrom	**1**	6,3
**Nebendiagnosen**		
Lungenarterienembolie	**0**	0,0
Tiefe Beinvenenthrombose	**1**	6,3
Thrombophlebitis	**9**	56,3
Koagulopathie	**3**	18,8

*MW* Mittelwert, *SD* Standardabweichung, *KTS* Klippel-Trénaunay-Syndrom, *CLOVE* Akronym für „congenital lipomatous overgrowth“, vaskuläre Fehlbildung und epidermaler Nävus, *BRBNS* „blue rubber bleb-naevus“, *CMTC* Cutis marmorata teleangiectatica congenita

**Table 2:** 

Klinische Symptomatik bei initialer Vorstellung	(*n*)	(%)
*Seite*		
Rechts	**10**	62,5
Links	**5**	31,3
Beidseits	**1**	6,3
*Beinlängendifferenz*	**12**	75,0
Beinverkürzung	**7**	43,8
Beinverlängerung	**4**	25,0
Verkürzung und Verlängerung	**1**	6,3
*Symptomatik*		
Weichteilhyperthrophie	**6**	37,5
Naevus flammeus	**13**	81,3
Stattgehabtes Erysipel	**3**	18,8
Ödeme	**8**	50,0
Schmerzen	**5**	31,3
Stattgehabte Blutungen	**5**	31,3
CVI	**12**	75,0
Ulcus cruris	**1**	6,3
AV-Fistel	**2**	12,5

*CVI* chronisch-venöse Insuffizienz, *AV* arteriovenöse Fistel

**Figure 2:**
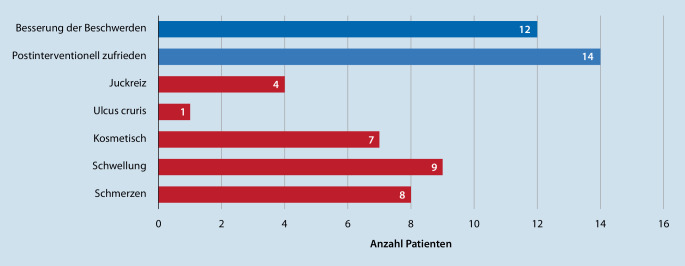

Insgesamt wurden 62,5 % (10/16) der Patienten zum Diagnosezeitpunkt mittels Kompression konservativ behandelt. Bei weiteren 31,3 % (5/16) Patienten erfolgte primär eine offen-chirurgische Entfernung der MV und bei einem der 16 Patienten wurde die MV primär mittels endovenöser Lasertherapie (EVLT) verschlossen. Fünfzehn Patienten wurden sekundär therapiert. Bei 2 der 15 Patienten erfolgte sekundär die offen-chirurgische Sanierung. Sechs der 15 Patienten wurden sekundär mittels EVLT sowie 7 mittels Sklerosierung therapiert. Pro Patient wurden 2,6 ± 2,4 (MW ± SD) Sekundärprozeduren durchgeführt. Bezogen auf das primäre Therapieverfahren gab es keinen signifikanten Unterschied in Bezug auf die Anzahl der Sekundäreingriffe (*p* = 0,058; Tab. 3). Zwölf der 16 Patienten gaben nach Intervention eine Besserung der Beschwerden an; 14 Patienten waren mit dem postinterventionellen Ergebnis zufrieden; 7 Patienten hatten postinterventionell eine anhaltende Schwellung der Extremität; 50 % (8/16) gaben postinterventionell weiterhin Schmerzen an (Abb. 2). Das mittlere Follow-up lag bei 8,1 Jahren (Tab. 3).

**Table 3:** 

	Total		Chirurgisch		Minimal-invasiv		*p*-Wert
Parameter	(*n*)	(%)	(*n*)	(%)	(*n*)	(%)	
*Patienten*	**15**	100	**6**	40,0	9	60	–
Anzahl Sekundärprozeduren (MW ± SD)	2,6 ± 2,4		1,4 ± 1,3		3,2 ± 2,7		0,058^a^
30-Tage-Mortalität nach Intervention	0 %						–
Follow-up (Jahre)	8,1						–

*MW* Mittelwert, *SD* Standardabweichung, *minimal-invasiv* mittels endovenöser Lasertherapie oder Sklerosierung

^a^Mann-Whitney-U-Test

## Chirurgische Entfernung der MV

In unserer chirurgischen Klinik haben sich Standards im operativen Vorgehen bei Therapie der MV etabliert, die wir im Folgenden beschreiben möchten.

Der Eingriff erfolgt in Allgemeinanästhesie und Rückenlagerung. Die Seitenäste und falls vorhanden AV-Fisteln werden im Rahmen der präoperativen Vorbereitung duplexsonographisch assistiert markiert. Nach sterilem Abwaschen und beweglichem Abdecken der betroffenen Extremität erfolgt die Freilegung der proximalen Einmündung der MV in die tiefe Vene und Ligatur der selbigen. Im Anschluss wird die markierte Vene über mehrere Hilfsschnitte von ca. 3 cm Länge freigelegt. Zwischen den einzelnen Inzisionen sollten Hautbrücken bestehen bleiben, um die Seitenäste und Fisteln direkt versorgen zu können. Die Marginalvene wird distal auf Höhe der Markierung ligiert. Wegen der Gefahr von Wundheilungsstörungen versuchen wir, den Fußrücken auszusparen. Jede Inzision wird schichtweise mit einer fortlaufenden Subkutannaht und einer intrakutan resorbierbaren Hautnaht verschlossen.

Ein klassisches Stripping der Vene nach Babcock ist bei der MV nicht empfohlen, da durch die kaliberstarken Seitenäste und AV-Fisteln starke Blutungen auftreten können. Die Marginalvene sollte zur Verhinderung von Rezidiven komplett entfernt werden.

**Figure 3:**
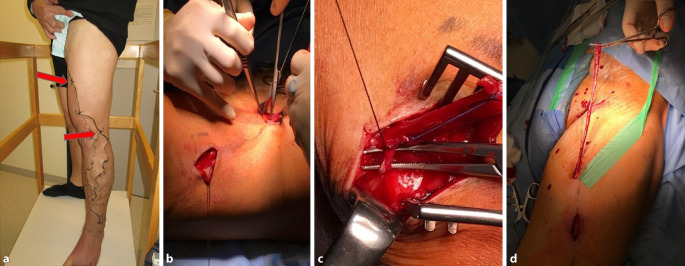

Postoperativ erhalten die Patienten eine standardisierte Thromboseprophylaxe mit niedermolekularem Heparin (subkutan appliziert) und einen oberschenkellangen Kompressionsstrumpf der Klasse II. Eine stationäre Überwachung für mindestens eine Nacht halten wir für obligat. Am 1. postoperativen Tag erfolgt eine klinische Kontrolle und wenn möglich die Entlassung. Eine Woche später erfolgt die ambulante Kontrolle. Das chirurgische Vorgehen ist in Abb. 3 dargestellt.

## Diskussion

Die Altersverteilung zum Diagnosezeitpunkt lag zwischen 3 und 42 Jahren bei einem mittleren Alter von 16,8 Jahren. Die Geschlechterverteilung war ausgeglichen. Dies korreliert mit den Angaben aus der Publikation von Weber. Hier zeigte sich ebenfalls ein mittleres Durchschnittsalter von 16,8 Jahren. Der Anteil der männlichen Patienten lag bei 53 % [26]. Auffällig ist, dass das Alter zum Diagnosezeitpunkt stark variiert. Eine mögliche Erklärung könnte die Seltenheit der Erkrankung und damit die Gefahr des Nichterkennens sein. Über den hohen Stellenwert einer frühzeitigen Diagnosestellung wurde bereits in mehreren Publikationen berichtet [9, 14]. Die Notwendigkeit einer frühzeitigen Diagnosestellung wird durch das Vorliegen weiterer behandlungsbedürftiger Symptome unterstrichen. Dabei ist insbesondere auf das kombinierte Vorliegen kongenitaler Syndrome zu achten [5, 23, 26]. Diese konnte auch bei 50 % der Patienten in unserem Kollektiv nachgewiesen werden. Das kombinierte Auftreten einer MV mit z. B. einem Klippel-Trénaunay-Syndrom wurde bereits in der Literatur beschrieben [5]. Das Klippel-Trénaunay-Syndrom ist gekennzeichnet durch das Vorliegen eines Naevus flammeus, der Hypertrophie einer Extremität sowie dem Vorliegen einer venösen Malformation [21].

Weiterhin lag bei 75 % (12/16) unserer Patienten eine Beinlängendifferenz vor. Es traten sowohl Beinlängenverkürzungen als auch Beinlängenverlängerungen auf (Tab. 2).

Warum eine MV gehäuft mit einer Beinlängendifferenz einhergeht, ist nicht abschließend geklärt. Als möglicher Auslöser einer Beinlängenverlängerung wird eine steigerte Perfusion der Wachstumsfugen in Folge des venösen Refluxes und dem gehäuften Vorhandensein von AV-Fisteln diskutiert [25]. Grundsätzlich muss eine Beinlängendifferenz frühzeitig erkannt werden, um entsprechende Behandlungsmaßnahmen einzuleiten. Matassi et al. favorisieren als primäre Therapie der Beinlängendifferenz eine frühzeitige, im Alter zwischen 5 und 8 Jahren durchgeführte chirurgische Versorgung der MV [23]. Weitere Behandlungsstrategien können von einfachen ausgleichenden Schuherhöhungen bis hin zur Durchführung einer Epiphyseodese reichen [11, 13]. Weiterhin kann durch die frühzeitige Intervention eine Progression der CVI verhindert und somit Spätfolgen einer CVI vermieden werden [3, 7, 14]. Nur bei 3 Patienten erfolgte die Diagnosestellung vor dem 8. Lebensjahr. Dies unterstreicht die Notwendigkeit, dem Auftreten einer atypischen Vene an der lateralen unteren Extremität Bedeutung zukommen zu lassen und eine entsprechende Behandlung einzuleiten. Da die Marginalvene auch als „Drainage“ ins tiefe Leitvenensystem dient, ist ein Verschluss dieser Vene zur Prophylaxe von Thrombembolien und Lungenembolien erforderlich [12]. Zwölf der 16 Patienten hatten zum Diagnosezeitpunkt eine klinisch relevante CVI. Aufgrund der überwiegend späten Diagnosestellung kann davon ausgegangen werden, dass die klinischen Beschwerden der MV mit nachfolgender CVI bereits bei diesen Patienten seit längerem bestanden, nicht mittels konservativer Verfahren aufgehalten werden konnten und die Patienten mit Einschränkungen in ihrem Alltag leben mussten [14]. Dazu gehören insbesondere die Neigung zu Ödemen, Schweregefühl und Ausbildung einer CVI mit hohem Schweregrad und Auftreten von Ulzerationen [2, 3, 7, 14]. Ein weiterer Grund für die nicht durchgeführte Therapie könnte in der Tatsache begründet sein, dass bei diesen Patienten eine AV-Fistel vorlag und gemäß der Publikation von Vollmar [9] das Vorhandensein einer AV-Fistel eine Kontraindikation zur chirurgischen Therapie einer MV darstellte. Dieser konservative Therapieansatz wurde inzwischen jedoch verlassen. Das Vorliegen einer AV-Fistel stellt heute keine Kontraindikation zur chirurgischen Sanierung mehr dar [23]. Wichtig ist hierbei die operative Vorgehensweise gemäß der oben beschriebenen „Step-by-step“-Technik (siehe oben).

Eine AV-Fistel lag in unserem Kollektiv bei 25 % der Patienten vor und damit deutlich unter den beschrieben 49 % von Weber [26]. Eine mögliche Ursache könnte sein, dass im Kollektiv von Weber in 49 % der Fälle mittels Angiographie eine AV-Fistel nachgewiesen wurde. In unserem Kollektiv erfolgte eine invasive Diagnostik nur in 2 Fällen zur Beurteilung des tiefen Venensystems. Weiterhin zeigte sich eine Diskrepanz in der Patientenverteilung in Bezug auf die MV-Klassifikation. In unserem Kollektiv konnten 60 % der Patienten der Typ-II-Klassifikation nach Weber und nur 25 % der Typ III oder IV zugeordnet werden (Tab. 1). In der Publikation von Weber et al. 2006 wurden über 70 % der Patienten dem Typ III oder IV zugeordnet. Diese Diskrepanz liegt möglicherweise in der unterschiedlichen Größe der Patientenkollektive (97 vs. 16 Patienten). Eine Typänderung ist durch ausbleibende invasive Therapie nicht möglich. Die Wahrscheinlichkeit für das Auftreten einer CVI ist für die Typen III und IV erhöht [26]. In unserem Kollektiv zeigten 12 der 16 Patienten zum Zeitpunkt der Diagnosestellung eine CVI. Acht der 12 Patienten mit einer CVI konnten dabei dem Typ 2 nach Weber zugeordnet werden. Dies steht im Kontrast zur Beobachtung von Weber et al. [26].

**Figure 4:**
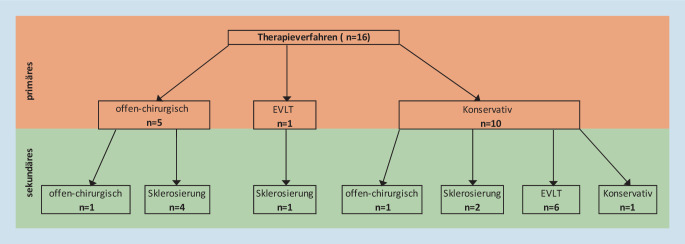

Die Therapie der Marginalvene unterliegt keinem kurativen Ansatz. Sie ist verbunden mit notwendigen Folgeeingriffen [3, 9]. Dies hat sich im vorgestellten Kollektiv bestätigt. Die mittlere Anzahl der notwendigen Sekundärprozeduren lag bei 2,6 ± 2,4 pro Patient (Tab. 3). Die primären und sekundären Therapierverfahren sind in Abb. 4 dargestellt. In diesem Zusammenhang sei darauf hingewiesen, dass mit einem mittleren Follow-up von 8,1 Jahren ein langfristiges Verlaufsintervall vorliegt. Bezogen auf die gesamte Lebensdauer kann zusätzlich von weiteren Eingriffen ausgegangen werden. Die unterschiedlichen Auswirkungen der Therapiestrategien auf das Outcome wurden bisher nicht hinreichend untersucht. In unserem Kollektiv zeigte sich kein signifikanter Unterschied zwischen den Therapieverfahren und den notwendigen Sekundäreingriffen (Tab. 3). Es zeigte sich jedoch eine Tendenz (*p* = 0,058), dass bei primär konventionell offen-chirurgisch versorgten Patienten weniger Sekundäreingriffe notwendig waren. Direkte Vergleiche mit der Literatur können hier nicht durchgeführt werden, da dies bisher nicht untersucht wurde.

Untersuchungen zur EVLT bei primärer Varikosis weisen Erfolgsraten zwischen 94–97 % auf [1, 15, 17, 20]. Einzelne Untersuchungen zur Anwendung der EVLT bei venösen Malformationen zeigen insbesondere für die Verbesserung der klinischen Symptomatik gute Ergebnisse [4, 24]. Die in der Literatur angegebenen Rezidivraten für die konventionell offen-chirurgische Therapie variieren zwischen 2,2 % und 60 % [6, 18] und sind abhängig vom Beobachtungszeitraum. Eine mögliche Ursache für das vermeintlich schlechtere Outcome der minimal-invasiven Therapien in unserer Studie könnte die durchgeführte Sklerotherapie bei 46,7 % der Fälle sein. Bei Rezidivraten von bis zu 15 % wird die Sklerotherapie zur Behandlung venöser Malformationen nicht empfohlen. Da Malformationen eine fuchsbauähnliche Struktur mit unregelmäßigen Gefäßdurchmessern aufweisen, ist eine suffiziente Verteilung des Therapeutikums schwierig zu erreichen [10]. Die Sklerotherapie wurde häufig als geplanter Folgeeingriff nach EVLT durchgeführt. Dies könnte eine mögliche Ursache für die tendenziell höheren Raten an Folgeeingriffen nach minimal-invasiver Therapie sein (Tab. 3).

Erfreulicherweise waren 14 von 15 Patienten nach Intervention (1 Patient wurde nicht interveniert) mit der durchgeführten Therapie zufrieden (Abb. 2). Zwölf der 16 Patienten gaben eine deutliche Besserung der Beschwerden an, 8 klagten über anhaltenden Schmerzen. In der Verlaufskontrolle berichteten 7 der 16 Patienten über kosmetische Probleme und 9 Patienten über anhaltende Beinschwellungen (Abb. 2). Die erhobenen Ergebnisse zeigen, dass die Therapie der Marginalvene mit einer Steigerung der Lebensqualität verbunden ist, jedoch keinem kurativem Ansatz unterliegt. Das frühzeitige Erkennen einer Marginalvene hat oberste Priorität, um Folgeerscheinungen und Schäden bei den betroffenen Patienten zu minimieren.

## Fazit

- Zur präoperativen Diagnostik eignen sich die FKDS und MR-Phlebographie. Bei Fragestellungen zur Aplasie/Hypoplasie ggf. die Phlebographie.
- Die invasive Behandlung der persistierenden MV geht mit einer Besserung der Beschwerdesymptomatik einher.
- Eine Progression der CVI konnte mittels konservativer Behandlung (Kompressionstherapie) nicht gestoppt werden.
- Zur Prävention/Vermeidung einer Progression einer CVI sollte nach Diagnosestellung die MV zeitnah verschlossen/entfernt werden.
- Durch die primäre Anwendung offen-chirurgischer Techniken scheinen Folgeeingriffen vermieden werden zu können.
- Es bedarf spezieller chirurgischer Techniken („Step-by-step“-Verfahren, kein Stripping mittels Sonde).
- Die Durchführung eines konsequenten Follow-ups zur Evaluation eventuell notwendiger Sekundäreingriffe und Vermeidung von Folgeschäden ist obligat.
